# Epigenetic clock analysis of human fibroblasts *in vitro*: effects of hypoxia, donor age, and expression of hTERT and SV40 largeT

**DOI:** 10.18632/aging.101955

**Published:** 2019-05-21

**Authors:** Mieko Matsuyama, David J. WuWong, Steve Horvath, Shigemi Matsuyama

**Affiliations:** 1 Division of Hematology and Oncology, Department of Medicine, School of Medicine, Case Western Reserve University, Cleveland, OH 44106, USA; 2 Department of Human Genetics and Biostatistics, David Geffen School of Medicine, University of California Los Angeles, Los Angeles, CA 90095, USA; 3 Case Comprehensive Cancer Center, Cleveland, OH 44106, USA

**Keywords:** epigenetic clock, epigenetic age, DNA methylation, hypoxia, immortalization

## Abstract

Aging is associated with a genome-wide change of DNA methylation (DNAm). "DNAm age" is defined as the predicted chronological age by the age estimator based on DNAm. The estimator is called the epigenetic clock. The molecular mechanism underlining the epigenetic clock is still unknown. Here, we evaluated the effects of hypoxia and two immortalization factors, hTERT and SV40-LargeT (LT), on the DNAm age of human fibroblasts in vitro. We detected the cell division-associated progression of DNAm age after >10 population doublings. Moreover, the progression of DNAm age was slower under hypoxia (1% oxygen) compared to normoxia (21% oxygen), suggesting that oxygen levels determine the speed of the epigenetic aging. We show that the speed of cell division-associated DNAm age progression depends on the chronological age of the cell donor. hTERT expression did not arrest cell division-associated progression of DNAm age in most cells. SV40LT expression produced inconsistent effects, including rejuvenation of DNAm age. Our results show that a) oxygen and the targets of SV40LT (e.g. p53) modulate epigenetic aging rates and b) the chronological age of donor cells determines the speed of mitosis-associated DNAm age progression in daughter cells.

## INTRODUCTION

There are two fundamental questions regarding the mechanism of aging. The first question is what kind of time-dependent changes (chemical and/or biological changes) drive aging. It is expected that there are factors other than time controlling the speed of aging since there is a variation of the lifespan and the timing of the onset of age-associated diseases. Therefore, the second question is what are these factors regulating the speed of aging. The present study investigates these two questions by focusing on the epigenetic aging of primary cultured human cells.

It is now widely accepted that there is an age associated change in DNAm in human as well as other species [1–5]. Most of the previously reported age-dependent DNAm changes were tissue- or cell type-specific (Reviewed in [1]). Recently, the pan-tissue age estimator was developed based on the DNAm levels of 353 CpG sites [3]. In 2018, another age estimator was developed that predicts the chronological age of skin and blood cells more accurately than the pan-tissue age estimator [6]. This new age estimator uses the DNAm levels of 391 CpG sites (60 CpG sites are overlapped with the pan-tissue age estimator) [6]. The DNAm-based age estimator are called the epigenetic clock, and the predicted age by the clock is called DNAm age [1]. The DNAm age functions as a biomarker to predict the risk of age-associated diseases as accelerated DNAm age has been observed in a variety of conditions, including obesity [7], HIV infection [8], Down syndrome [9], Parkinson's disease [10], Werner syndrome [11], and menopause [12].

The DNAm age of blood is predictive of lifespan, even after adjusting for chronological age and other risk factors [13, 14]. Lifestyle factors have only a weak effect on the DNAm age of blood [15], suggesting that DNAm age largely reflects cell-intrinsic properties. In a recent study, we tested this postulated cellular autonomy by measuring DNAm age in blood cells of leukemia patients after hematopoietic stem cell transfer (HSCT) from allogeneic donors [16]. We found that the DNAm age of the donor’s blood cells was not influenced by the recipient’s body, whether younger or older, and that the DNAm age progressed as if the donor’s cells were still in the donor’s body, even 17 years after HSCT [16]. This finding further supports that the epigenetic clock is a cell-intrinsic phenomenon at least in hematopoietic cells.

Overall, there is strong indirect evidence that the epigenetic clock relates to one or more innate aging processes. However, it has been challenging to dissect the molecular mechanisms underlying the epigenetic clock. To address this challenge, *in vitro* models may be used. Here, we used primary cultured human fibroblasts to carry out the epigenetic clock analysis of three conditions that relate to the replicative lifespan: hypoxia, donor age, and immortalization. The present results provide clues to identify molecular machineries controlling the epigenetic aging. Based on our findings in this study, we will discuss the hypothetical molecular mechanisms regulating the progression of the DNAm age.

## RESULTS

### Effects of hypoxia on cell proliferation

**Figure 1:**
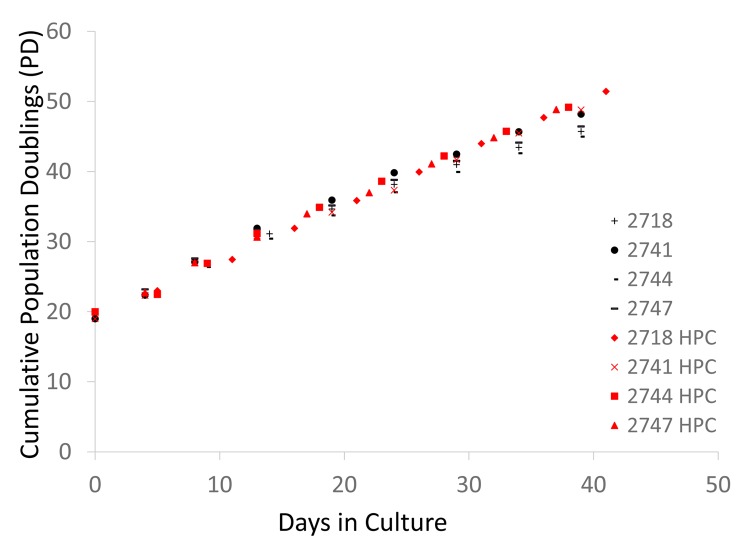
**Cell division records of neonate fibroblast cell lines cultured in normoxia and hypoxia.** Each 4-digit number in the graph (#2718, #2741, #2744, and # 2747) indicates the batch of the cell line from the vendor (Cell Applications, San Diego, CA). The graph shows the cumulative population doublings (PD) of each cell line in normoxia and hypoxia. Black and red symbols indicate the data from normoxia and hypoxia, respectively.

**Figure 2:**
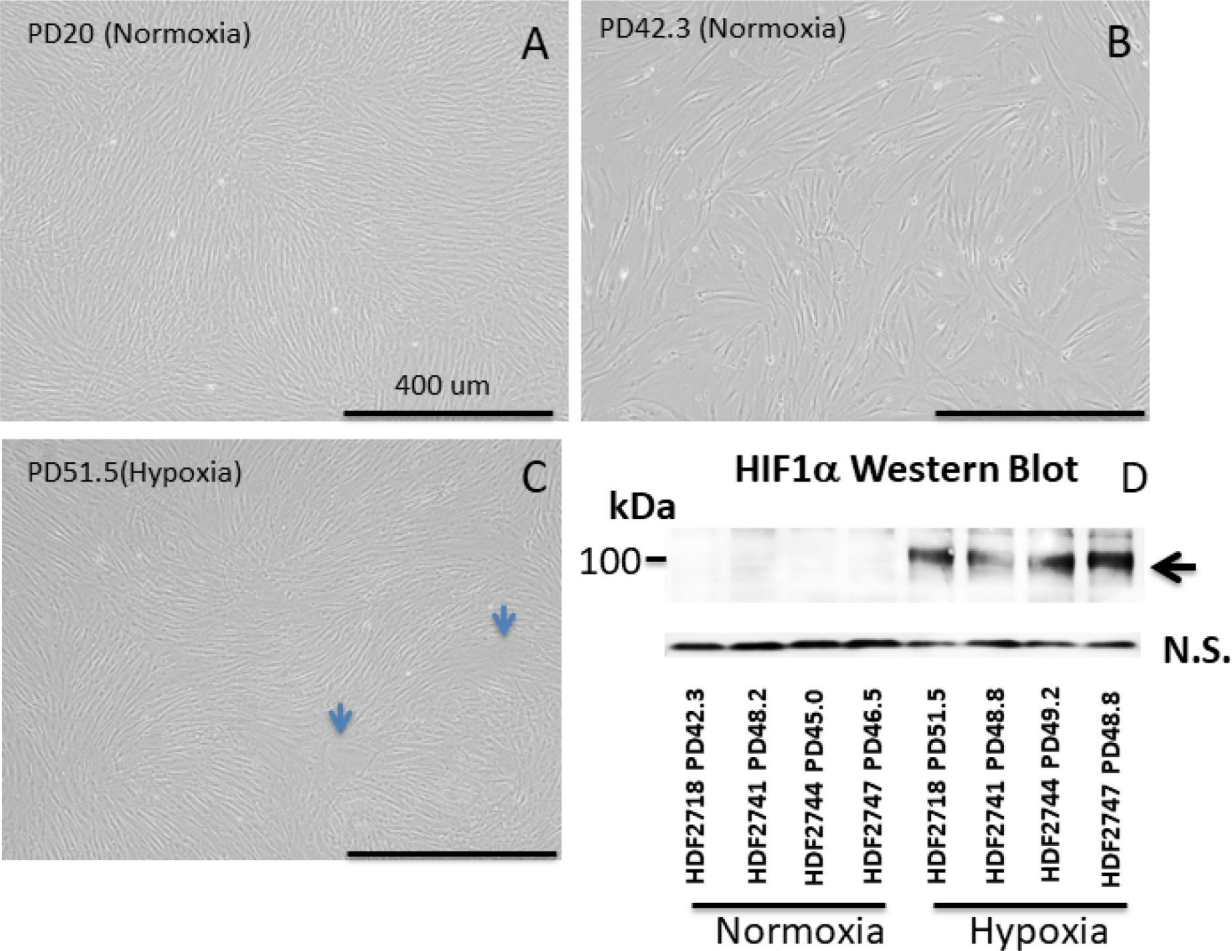
**Images of neonate human dermal fibroblasts (HDFs) (A–C) and Western blot of HIF1α (D).** (**A–C**) HDFs cultured in normoxia and hypoxia. All images were taken at the same magnification. Each 4-digit number in the graph (#2718, #2741, #2744, and # 2747) indicates the batch of the cell line from the vendor (Cell Applications, San Diego, CA). Bar: 400 μm. (**A**) HDFs at population doubling (PD) number 20 in normoxia, (**B**) HDFs at PD 42.3 in normoxia, (**C**) HDFs at PD 51.5 in hypoxia. Cells cultured in hypoxia maintained a small cell size in comparison with cells in normoxia. (**D**) Western blot of HIF1α. HIF1α was stabilized in all 4 cell lines cultured under hypoxia.

Human dermal fibroblasts from neonates were cultured under normoxic conditions in a CO_2_ incubator [5% CO_2_ and 21% O_2_ (atmospheric)] as well as under hypoxic conditions (5% CO_2_ and 1% O_2_). The cells were collected and passaged every 4-6 days. Excess cells that were not used for the passage were frozen at -80C for protein and DNA analysis. As previously established [17], in normoxia, the cell division speed declined after PD30 (Figure 1, black symbols), and the cell size increased (Figure 2A and B). In contrast, under hypoxia, the cells maintained a constant division speed after PD30 (Figure 1, red symbols), and the cells did not become as large as the cells under normoxia (Figure 1C), though a small proportion of cells did show size enlargement (arrows in Figure 2C). The stabilization of HIF1α (hypoxia-induced factor 1α) was confirmed in all four cell lines incubated under hypoxia (Figure 2D and Supplementary Figure 1).

### Effects of hypoxia on the progression of DNAm age

DNAm age was estimated by two methods that apply to in vitro studies: Horvath's pan tissue clock based on 353 CpGs [3] and the more recent skin & blood clock based on 391 CpG sites [6]. The corresponding DNAm age estimates will be referred to as DNAmAge^353CpG^ and DNAmAge^391CpG^, respectively. Previously, we reported that DNAmAge^391CpG^ predicts the chronological age of cultured fibroblasts more accurately than the original DNAmAge^353CpG^ [6]. The present study used both DNAmAge^353CpG^ and DNAmAge^391CpG^ so that the present results can be compared with previous studies.

**Figure 3:**
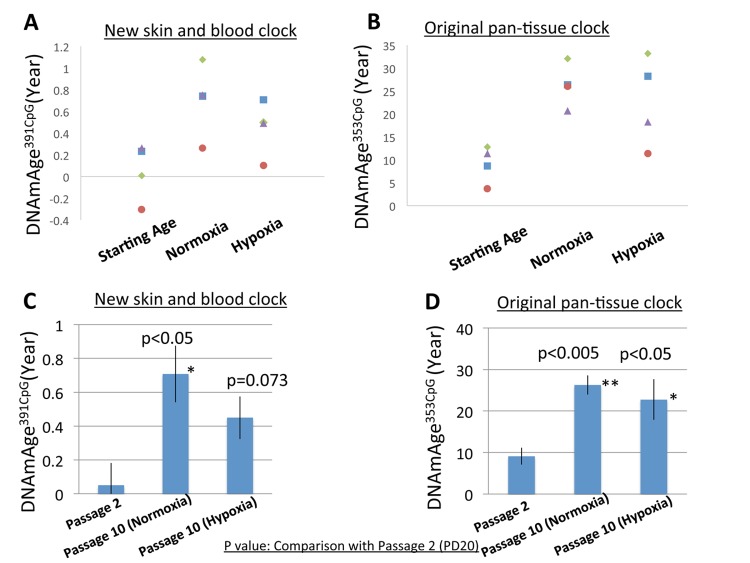
**DNAm age progression in normoxia and hypoxia.** (**A** and **B**) The DNAm age of each cell line at early PD (19-20) (passage 2) and accumulated PD (42.3-51.5) (passage 10) is shown. The DNAm age of the same cell line is shown by the same color. Results for DNAmAge^391CpG^ (**A**) and DNAmAge^353CpG^ (**B**) are shown. Each dot is the average of a duplicate DNAm analysis for each condition. (**C** and **D**) The average DNAm ages of 4 cell lines at passage 2, passage 10 (Normoxia), and passage 10 (Hypoxia) are shown (n=4). Results for DNAmAge^391CpG^ (**C**) and DNAmAge^353CpG^ (**D**) are shown. The P values on the figure show the statistical comparison with the data at passage 20. *p<0.05, **p<0.005 (t-test).

**Figure 4:**
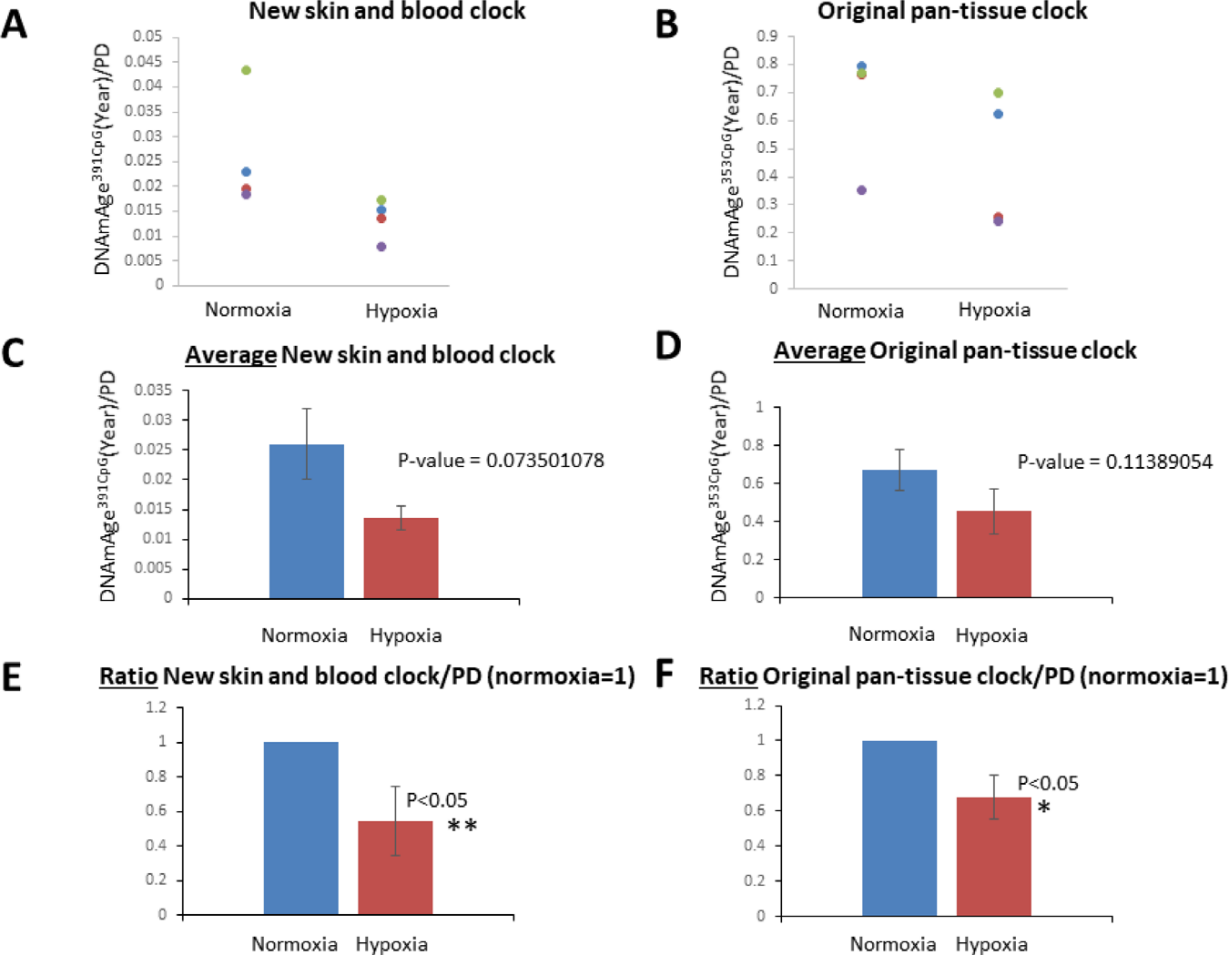
**Hypoxia slowed the speed of the cell division-associated DNAm age progression.** (**A** and **B**) DNAmAge^391CpG^/PD (**A**) and DNAmAge^353CpG^/PD (**B**) of each cell line in normoxia and hypoxia are shown. The result from the same cell line is marked with the same color. In all cell lines examined, hypoxia slowed the speed of DNAm age progression. (**C** and **D**) Average and S.E. of DNAmAge^391CpG^/PD and DNAmAge^353CpG^/PD are shown. (**E** and **F**) In these graphs, DNAm age/PD in normoxia is designated as 1 in all cell lines, and the ratio of DNAm age in normoxia and hypoxia was calculated. In both DNAmAge^391CpG^ and DNAmAge^353CpG^, hypoxia slowed the speed of the progression of DNAm age and the effects were statistically significant (t-test).

As shown in Figure 3, the progression of DNAmAge^391CpG^ (Figure 3A, 3C) and DNAmAge^353CpG^ (Figure 3B, 3D) was observed after eight passages, which is equal to an additional 22.3-31.5 population doublings (PD) (Figure 3A, 3B). These increases of DNAm age (both DNAmAge^391CpG^ and DNAmAge^353CpG^) were statistically significant in normoxia (*p<0.05, **p<0.005, Figure 3C and 3D), indicating that 6 weeks of cell culture is sufficient to detect progression of the DNAm age. As previously reported, DNAmAge^391CpG^ accurately predicted the donor’s age (0 years old for neonate cells) whereas DNAmAge^353CpG^ overestimated the donor’s age (4-12 years old for neonate cells) of the cultured fibroblasts (Figure 3B, 3D). Importantly, hypoxia slowed the progression of both DNAmAge^391CpG^ and DNAmAge^353CpG^ in all 4 cell lines examined (Figure 4A, 4B). The average values of DNAm age progression per PD are shown in Figure 4C and 4D. The effect of hypoxia on slowing DNAm age progression was statistically significant when contrasting the ratio of speeds (Figure 4E, 4F). Hypoxia allows fibroblasts to continue cell division by extending the replicative lifespan [17]. Similarly, we found that the fibroblasts were able to divide more under hypoxia than under normoxia (Figure 1). Nevertheless, the progression of DNAm age per cell division or PD diminished in the hypoxia group.

### Comparison of cell division-associated DNAm age progression between neonate and adult fibroblasts

**Figure 5:**
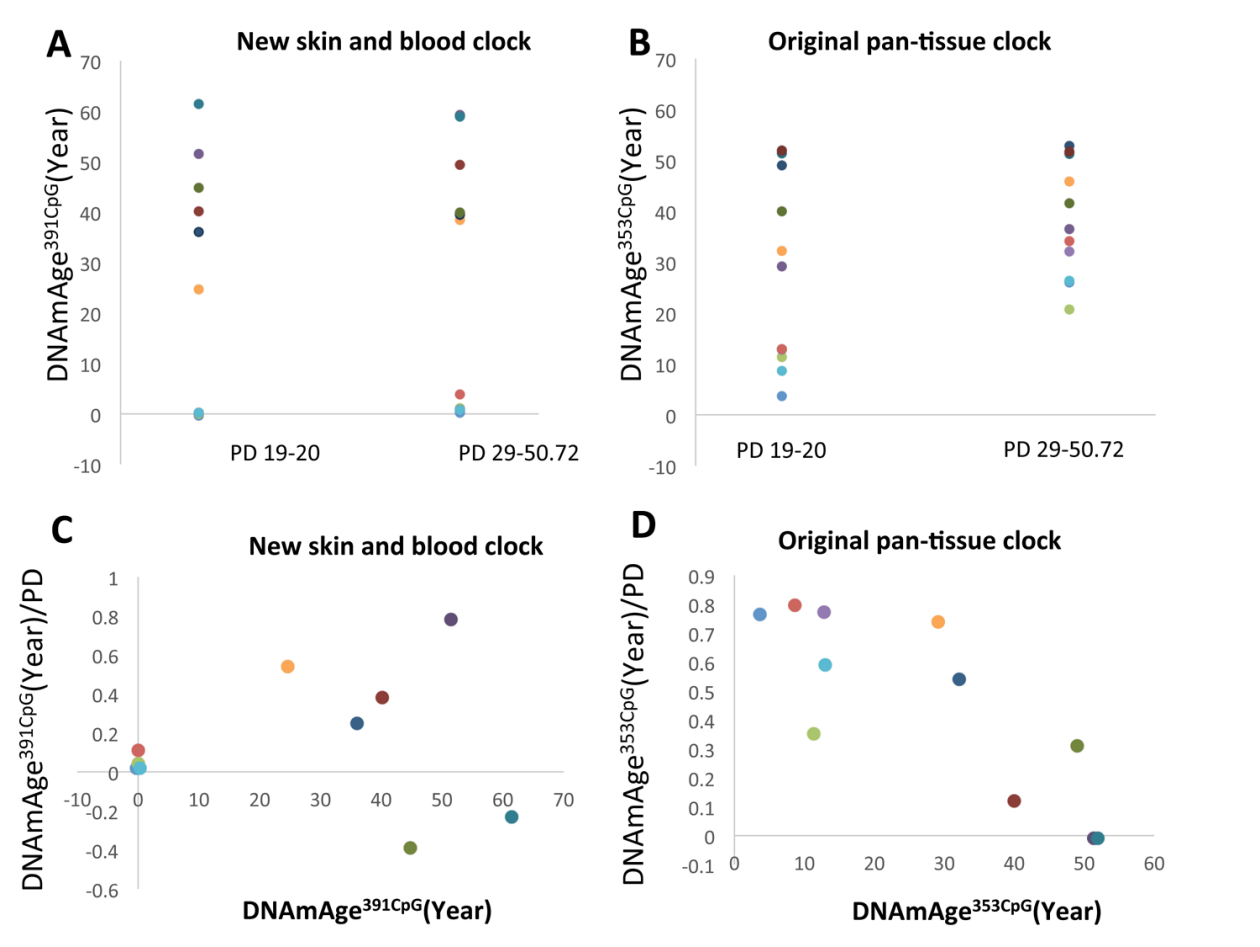
**The progression of DNAm age after more than 10 PD of fibroblasts from donors of different ages from.** (**A** and **B**) The progression of DNAmAge^391CpG^ (**A**) and DNAmAge^353CpG^ (**B**) after more than 10 PD of cell culture is shown. Each panel shows the following information: (**A** and **B**) Changes of DNAm age after more than 10 PD. (**C** and **D**) The Y axis indicates the values of DNAmAge^391CpG^/PD (**C**) and DNAmAge^353CpG^/PD (**D**). The X-axis indicates the DNAm age at the beginning of this experiment. The impact of PD on the speed of DNAm age progression becomes greater when the starting DNAmAge^391CpG^ is older, but becomes lesser when the starting DNAmAge^353CpG^ is older. The dots with the same color are the results from the same cell line.

In addition to fibroblasts from neonates, we also examined fibroblasts from adult donors. We used a total of 11 different fibroblast lines; 5 from neonates and 6 from adult donors (Figure 5). DNAmAge^391CpG^ and DNAmAge^353CpG^ progressed by cell division in the majority of adult cells (4 cell lines among 6 cell lines for DNAmAge^391CpG^ (Figure 5A) and 5 cell lines among 6 cell lines for DNAmAge^353CpG^ (Figure 5B)). However, the speed of DNAm age progression varied depending on the age of the cells. The cell division-associated progression of DNAmAge^353CpG^ became slower as the starting age became older (Figure 5B, 5D). In contrast, DNAmAge^391CpG^ showed a different trend. The progression of DNAmAge^391CpG^ became more significant when considering an older starting age (Figure 5A, 5C). Interestingly, 2 cases among 6 cases, DNAmAge^391CpG^ showed rejuvenation (light blue and green symbols in Figure 5C)]. At present, it is not certain whether this rejuvenation phenomenon is always reproducible at this rate (2 out of 6, i.e. 33%) even if we increase the sample number. Larger scale experiments sre needed to validate this phenomenon.

### Effects of the expression of hTERT or SV40LT on the DNAm age

**Figure 6:**
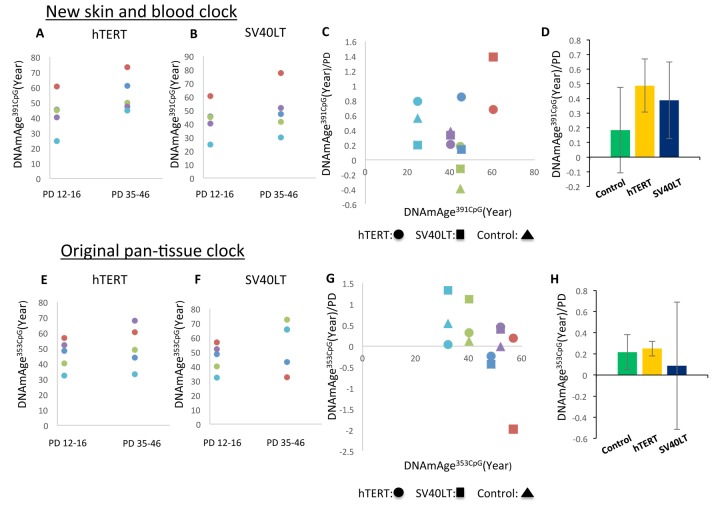
**Effects of hTERT and SV40LT transfection on the DNAm age of fibroblasts.** These panels show DNAmAge^391CpG^ (**A**–**D**) and DNAmAge^353CpG^ (**E**–**H**) of fibroblasts transfected with the hTERT and SV40LT genes, respectively. The changes of DNAm age more than 20 PD after hTERT (**A** and **E**) or SV40LT (**B** and **F**) gene transfection are shown. The dots with the same color are the results from the same cell line. Panels (**C** and **D**) show DNAmAge^391CpG^/PD (**C**) and DNAmAge^353CpG^/PD (**G**) of each cell line transfected with the hTERT or SV40LT gene. As references, the DNAm age/PD values of non-transfected cells are shown for 3 cell lines. The starting DNAm age values are shown on the X axis. Panels (**D** and **H**) show the average and S.E. of DNAmAge^391CpG^/PD (**D**) and DNAmAge^353CpG^/PD (**H**) of non-transfected control cells (n=3), hTERT (n=5), and SV40LT (n=5). The effects of SV40LT were not consistent, and the S.E. of the average is very large.

Next, we examined the effects of two commonly used immortalization methods: hTERT and SV40LT expression (reviewed in [18]). Retrovirus vectors were used to introduce the hTERT or SV40LT gene into the primary cultured fibroblasts as previously described [19, 20]. Retrovirus infection was performed at passage 2 (PD10-15), and the cells were cultured for more than 6 additional passages. We examined 5 different primary cultured fibroblast cell lines obtained from 5 different adult donors. As shown in Figure 6A and 6C, hTERT expression resulted in continuous progression of DNAmAge^391CpG^ in all cell lines examined. In the case of DNAmAge^353CpG^ analysis, hTERT expression maintained the progression of DNAmAge in 4 of 5 cell lines (Figure 6E, 6G), as previously reported [6, 21]. On the other hand, the effects of SV40LT were inconsistent: DNAmAge^391CpG^ and DNAmAge^353CpG^ progression was maintained or reversed depending on the cell line (Figure 6B, 6C (DNAmAge^391CpG^) and Figure 6F, 6G (DNAmAge^353CpG^)). For example, SV40LT expression resulted in the rejuvenation (decrease) of DNAmAge^353CpG^ in 2 cell lines after several passages (Figure 6G, dots below the line of 0 year/PD). In Figure 6D and 6H, the mean and S.E. of the speed of DNAm age progression per PD is shown. There were no significant differences (control, hTERT or SV40LT expression). The extremely large S.E. of the SV40LT group in Figure 6H reflects the inconsistent (acceleration or rejuvenation) effects on DNAmAge^353CpG^, mentioned previously.

## DISCUSSION

The present study demonstrated that (1) hypoxia slows down the progression of DNAm age, (2) cell division-associated DNAm age progression depends on the chronological age of the cell donor, (3) SV40LT immortalization induces abnormal progression of DNAm age, and (4) hTERT expression does not arrest progression of DNAmAge.

The present study showed that DNAm age of fibroblasts progressed after multiple cell divisions *in vitro*. In this study, neonate fibroblasts were cultured approximately 6 weeks with 8 passages (4-6 days per passage). This means that 6 weeks is long enough to detect the progression of DNAm age in cell culture experiments. However, the speed of DNAm age progression in cell culture was much faster than the chronological time. For example, approximately 0.65 years [Figure 3C; from 0.05 ± 0.131 (passage 2) to 0.708 ± 0.168 (passage 8)] of DNAmAge^391CpG^ progressed over 6 weeks (equivalent to 0.12 years) under normoxia (Figure 3C); that is, the speed of DNAmAge^391CpG^ progression was 5 times faster than the chronological time. Interestingly, this speed became slower under hypoxia; 0.4 years [Figure 3C; from 0.05 ± 0.131 (passage 2) to 0.450 ± 0.126 (passage 8)] of DNAmAge^391CpG^ progression for 6 weeks (0.12 years) (Figure 3C). The oxygen concentration in the human body is much lower than that of the air (21% oxygen). For example, the oxygen concentration of the brain, liver and kidney is reported to be approximately 2-3% and that of the hematopoietic stem cell niche is approximately 1-2% [22, 23]. One of the reasons for the fast progression of the DNAm age in the conventional cell culture may be the high oxygen concentration (normoxia) in the medium. The present study suggests that oxygen is a factor increases the epigenetic aging, a finding which could elucidate the molecular mechanism of the epigenetic clock. It is well established that Hypoxia-Inducible Factor 1α (HIF1α, a transcriptional factor), mediates various cellular responses to hypoxia [24]. HIF1α is reported to decrease expression levels of DNA methyl-transferases (DNMTs: DNMT1, 3A and 3B), whereas it increases expression of DNA de-methylatiing enzymes (Ten-eleven Translocation (TET) 2 and 3) [24]. Thus, HIF1α -induced modulations of the balance of DNMTs and TETs is a plausible mechanism of how hypoxia slows down the epigenetic clock.

In addition to oxygen, there are other factors in conventional cell culture systems that could potentially impact the epigenetic clock. For example, the fibroblast culture medium (FGM) used in this study contains fetal calf serum (FCS) and 8 mM glucose, which could be the reasons of the accelerated epigenetic aging of cells in the conventional cell culture system. Further investigation of the effects of these and other constituents of cell culture systems is warranted to identify the factors accelerating the progression of DNAm age.

In this study, we examined fibroblasts from donors of various ages. Neonate cells progressed their DNAmAge^353CpG^ at a higher rate than adult cells did. As shown in Figure 5C, 5D, the progression per each cell division became less when the starting age was older. This result suggests that mitosis is accounted as a factor progressing DNAmAge^353CpG^ when the human body is growing (e.g. neonate), but it becomes less critical after the human body has reached maturity. In the case of DNAmAge^391CpG^, 67% (4 cell lines among 6 cell lines) of adult cell lines (>25 years old) showed faster progression than neonate cells. This means that older cells are more sensitive to cell division in terms of progression of DNAmAge^391CpG^ (Figure 5B). Since DNAmAge^391CpG^ predicts the chronological age of fibroblasts more accurately than DNAmge^353CpG^, we speculate that the observation based on DNAmAge391CpG represent the significance of cell division in the epigenetic aging process of fibroblasts. Although the two epigenetic age estimators showed different behaviors, the common point is that the speed of the cell division-associated progression of DNAm age was found to be different between neonate and adult cells. The present result suggests that the age of mother cells is a factor controlling the speed of mitosis-associated progression of the DNAm age in daughter cells.

The age-dependent increases of the methylation of CpGs in KLF14 gene locus (KLF14-CpGs) has been reported in human [25, 26] as well as mouse and monkey [27]. KLF14-CpGs are included in the two age estimator programs used in this study [3, 6]. Recently, it was found that hypermethylation of KLF14-CpGs is associated with the decrease of DNMT1 gene expression in human fibroblasts [28]. There is another study reporting the age-dependent decrease of the gene expression of DNMT1 and DNMT3B in human peripheral mononuclear cells [29]. The present study showed that mitosis-associated progression of DNAm age was influenced by the age of the cell. This observation may be explained by the change of the expression levels of DNMTs that is linked with the age-dependent hypermethylation of KLF14-CpGs.

We examined the effects of two immortalization treatments on the epigenetic age of cultured cells: expression of hTERT and SV40LT. In the case of hTERT expression, DNAmAge^391CpG^ increased in all 5 cell lines examined (Figure 6A, 6C) and DNAmAge^353CpG^ increased in 4 of the 5 of cell lines (Figure 6E, 6G). This is consistent with previous reports using keratinocytes, fibroblasts and endothelial cells [6, 21]. In the case of DNAmAge^353CpG^, one of the 5 cell lines showed a slight decrease (Figure 6E dark blue dots). At present, we do not have a clear explanation of this unexpected result. However, one of the technical pitfalls of retrovirus-mediated transfection is the uncontrolled integration of the transfected gene into the host chromosomes. Thus, chromosomal position effects and copy number variations related to the integrated hTERT gene might lead to differences in DNAm age among transfected cell lines.

For certain cancer types, p53 mutations have been associated with *slower* epigenetic age acceleration in malignant tissue samples [3, 30]. Horvath (2013) hypothesized that p53 might be part of an epigenomic maintenance system that underlies epigenetic aging. Our *in vitro* studies of SV40LT (which is a well-known p53 inhibitor) support such a role for p53 in maintaining the normal progression of the epigenetic clock. Specifically, we found that SV40LT had inconsistent effects on both DNAmAge^391CpG^ and DNAmAge^353CpG^. These data support the hypothesis that p53 inactivation induces malfunction of the cellular system governing the process of epigenetic aging. As a caveat, SV40LT also perturbs other genes besides p53. It is known to perturb the retinoblastoma family of tumor suppressors and pp2A phosphatase [31, 32]. To further determine the role of p53 in the regulation of the epigenetic aging, the specific inhibition of p53 by siRNA or targeted mutation of p53 by CRISPR/Cas9, for examples, will be necessary.

The present study suggests that mitosis-associated progression of the epigenetic clock is under the influence of oxygen level, the age of the cell, and tumor suppressors. Although cell division progresses the DNAm age, its speed is expected to be very different depending on the differentiation status and other environmental conditions. It is because the multiple cell types with different proliferation history show a similar DNAm age which is very close to the chronological age of the whole body. Our finding indicates that oxygen, the age, and tumor suppressors are the candidates of these factors controlling the speed of the epigenetic clock in different tissues. Further studies are necessary to investigate how multiple cell types in a human body can progress the DNAm age at the similar speed as the chronological age progresses.

## METHODS

### Epigenetic clocks

Several DNAm-based biomarkers have been proposed in the literature which differ in terms of their applicability (some are developed for specific tissues such as blood) and in terms of their biological interpretation. In this study, we focused on two epigenetic clocks that apply to fibroblasts. The pan-tissue epigenetic clock (Horvath 2013) is based on 353 CpGs. It applies to all sources of DNA (with the exception of sperm). The resulting age estimate (in units of years) is referred to as DNAmAge^353CpG^. Different postmortem tissues collected from the same individual have roughly the same epigenetic age [3, 6]. Hence, the pan tissue clock does not measure proliferative history (or mitotic age) in most cell types and tissues. We recently developed a new epigenetic age estimator (referred to as the skin and blood clock or DNAmAge^391CpG^) that leads to substantially more accurate age estimates in fibroblasts, keratinocytes, buccal cells, blood cells, saliva, and endothelial cells [3].

### DNA extraction and methylation analysis

DNA was extracted and purified from 2-3 million cells/sample by using a QIAGEN DNA extraction kit (Cat #51304) according to the manufacturer’s protocol. To remove RNA, RNAase (ThermoFisher Cat# EN0531, final 100 μg/ml) was added to the cell suspension buffer during the DNA extraction procedure. DNA methylation levels were measured on Illumina 450K arrays according to the manufacturer’s instructions. The Illumina BeadChip (450K) measures bisulfite-conversion-based, single-CpG resolution DNAm levels at different CpG sites in the human genome. These data were generated by following the standard protocol for Illumina methylation assays, which quantifies methylation levels by the β value using the ratio of intensities between methylated and un-methylated alleles. Specifically, the β value is calculated from the intensity of the methylated (M corresponding to signal A) and un-methylated (U corresponding to signal B) alleles, as the ratio of fluorescent signals β = Max(M,0)/[Max(M,0)+ Max(U,0)+100]. Thus, β values range from 0 (completely un-methylated) to 1 (completely methylated). We used the "noob" normalization method, which is implemented in the "minfi" R package [33, 34].

### Cell culture

Human dermal fibroblasts were obtained from two different sources, Cell Applications (San Diego, CA) and the Progeria Research Foundation (PRF). The cells from PRF are from healthy donors who did not have Progeria-related mutations. Fibroblasts were obtained as a frozen stock, and these cells had already reached 9-10 population doublings. Fibroblasts were cultured using Human Fibroblast Growth (HGF) Medium (Cell Applications) that contains 8 mM glucose and 2% FCS as a supplement. The cell density was 0.2 million cells/15 ml/dish at the beginning of each passage culture. When cells reached the confluent condition after 4-6 days, they were collected and the total cell number was determined. At each passage stage, unused cells were frozen for DNAm analysis and HIF1α protein expression. To examine the effects of hypoxia, cells were cultured in an hypoxia chamber (1% O2) as explained in the Results section. 

### hTERT and SV40Large T expression

hTERT or SV40LT (Large T) was expressed using retrovirus-mediated gene transfection according to previously established methods [19, 20]. The plasmids necessary for retrovirus synthesis were the kind gifts from Dr. Mark Jackson (Pathology, Case Western Reserve University).

### Western blot

Cell pellets were lysed in RIPA buffer (20 times pipetting and 20 min rotation at 4°C), and the soluble fraction was collected after centrifugation at 14,000 rpm for 30 min. Samples with 20 μg total protein was loaded in each lane. HIF1α antibody and the secondary antibody (anti-rabbit goat IgG-HRP) were purchased from NOVUS Biologicals (Cat # NB100-479) and DAKO (Cat # P0448), respectively. Chemiluminescence substrate was purchased from Kindle Biosciences (Cat #R1004) (www.kindlebio.com). Western blot image was obtained by using a digital camera (KwicQuant Imager, Kindle Biosciences).

## Supplementary Material

Supplementary Figure

## References

[1] Horvath S, Raj K. DNA methylation-based biomarkers and the epigenetic clock theory of ageing. Nat Rev Genet. 2018; 19:371–84. 10.1038/s41576-018-0004-3 29643443

[2] Hannum G, Guinney J, Zhao L, Zhang L, Hughes G, Sadda S, Klotzle B, Bibikova M, Fan JB, Gao Y, Deconde R, Chen M, Rajapakse I, et al. Genome-wide methylation profiles reveal quantitative views of human aging rates. Mol Cell. 2013; 49:359–67. 10.1016/j.molcel.2012.10.016 23177740 PMC3780611

[3] Horvath S. DNA methylation age of human tissues and cell types. Genome Biol. 2013; 14:R115. 10.1186/gb-2013-14-10-r115 24138928 PMC4015143

[4] Spólnicka M, Piekarska RZ, Jaskuła E, Basak GW, Jacewicz R, Pięta A, Makowska Ż, Jedrzejczyk M, Wierzbowska A, Pluta A, Robak T, Berent J, Branicki W, et al. Donor age and C1orf132/MIR29B2C determine age-related methylation signature of blood after allogeneic hematopoietic stem cell transplantation. Clin Epigenetics. 2016; 8:93. 10.1186/s13148-016-0257-7 27602173 PMC5012039

[5] Weidner CI, Wagner W. The epigenetic tracks of aging. Biol Chem. 2014; 395:1307–14. 10.1515/hsz-2014-0180 25205717

[6] Horvath S, Oshima J, Martin GM, Lu AT, Quach A, Cohen H, Felton S, Matsuyama M, Lowe D, Kabacik S, Wilson JG, Reiner AP, Maierhofer A, et al. Epigenetic clock for skin and blood cells applied to Hutchinson Gilford Progeria Syndrome and ex vivo studies. Aging (Albany NY). 2018; 10:1758–75. 10.18632/aging.101508 30048243 PMC6075434

[7] Horvath S, Erhart W, Brosch M, Ammerpohl O, von Schönfels W, Ahrens M, Heits N, Bell JT, Tsai PC, Spector TD, Deloukas P, Siebert R, Sipos B, et al. Obesity accelerates epigenetic aging of human liver. Proc Natl Acad Sci USA. 2014; 111:15538–43. 10.1073/pnas.1412759111 25313081 PMC4217403

[8] Horvath S, Levine AJ. HIV-1 Infection Accelerates Age According to the Epigenetic Clock. J Infect Dis. 2015; 212:1563–73. 10.1093/infdis/jiv277 25969563 PMC4621253

[9] Horvath S, Garagnani P, Bacalini MG, Pirazzini C, Salvioli S, Gentilini D, Di Blasio AM, Giuliani C, Tung S, Vinters HV, Franceschi C. Accelerated epigenetic aging in Down syndrome. Aging Cell. 2015; 14:491–95. 10.1111/acel.12325 25678027 PMC4406678

[10] Horvath S, Ritz BR. Increased epigenetic age and granulocyte counts in the blood of Parkinson’s disease patients. Aging (Albany NY). 2015; 7:1130–42. 10.18632/aging.100859 26655927 PMC4712337

[11] Maierhofer A, Flunkert J, Oshima J, Martin GM, Haaf T, Horvath S. Accelerated epigenetic aging in Werner syndrome. Aging (Albany NY). 2017; 9:1143–52. 10.18632/aging.101217 28377537 PMC5425119

[12] Carroll JE, Irwin MR, Levine M, Seeman TE, Absher D, Assimes T, Horvath S. Epigenetic Aging and Immune Senescence in Women With Insomnia Symptoms: Findings From the Women’s Health Initiative Study. Biol Psychiatry. 2017; 81:136–44. 10.1016/j.biopsych.2016.07.008 27702440 PMC5536960

[13] Chen BH, Marioni RE, Colicino E, Peters MJ, Ward-Caviness CK, Tsai PC, Roetker NS, Just AC, Demerath EW, Guan W, Bressler J, Fornage M, Studenski S, et al. DNA methylation-based measures of biological age: meta-analysis predicting time to death. Aging (Albany NY). 2016; 8:1844–65. 10.18632/aging.101020 27690265 PMC5076441

[14] Marioni RE, Shah S, McRae AF, Chen BH, Colicino E, Harris SE, Gibson J, Henders AK, Redmond P, Cox SR, Pattie A, Corley J, Murphy L, et al. DNA methylation age of blood predicts all-cause mortality in later life. Genome Biol. 2015; 16:25. 10.1186/s13059-015-0584-6 25633388 PMC4350614

[15] Quach A, Levine ME, Tanaka T, Lu AT, Chen BH, Ferrucci L, Ritz B, Bandinelli S, Neuhouser ML, Beasley JM, Snetselaar L, Wallace RB, Tsao PS, et al. Epigenetic clock analysis of diet, exercise, education, and lifestyle factors. Aging (Albany NY). 2017; 9:419–46. 10.18632/aging.101168 28198702 PMC5361673

[16] Sorras AM, Dahl A, Horvath S, Matsuyama S. Epigenetic Age is a Cell-Intrinsic Property in Transplanted Human Hematopoietic Cells. Aging Cell. 2019; 18:e12897. 10.1111/acel.12897 30712319 PMC6413751

[17] Welford SM, Bedogni B, Gradin K, Poellinger L, Broome Powell M, Giaccia AJ. HIF1alpha delays premature senescence through the activation of MIF. Genes Dev. 2006; 20:3366–71. 10.1101/gad.1471106 17142669 PMC1698444

[18] Campisi J, d’Adda di Fagagna F. Cellular senescence: when bad things happen to good cells. Nat Rev Mol Cell Biol. 2007; 8:729–40. 10.1038/nrm2233 17667954

[19] Hahn WC, Counter CM, Lundberg AS, Beijersbergen RL, Brooks MW, Weinberg RA. Creation of human tumour cells with defined genetic elements. Nature. 1999; 400:464–68. 10.1038/22780 10440377

[20] Zhao JJ, Gjoerup OV, Subramanian RR, Cheng Y, Chen W, Roberts TM, Hahn WC. Human mammary epithelial cell transformation through the activation of phosphatidylinositol 3-kinase. Cancer Cell. 2003; 3:483–95. 10.1016/S1535-6108(03)00088-6 12781366

[21] Kabacik S, Horvath S, Cohen H, Raj K. Epigenetic ageing is distinct from senescence-mediated ageing and is not prevented by telomerase expression. Aging (Albany NY). 2018; 10:2800–15. 10.18632/aging.101588 30332397 PMC6224244

[22] Carreau A, El Hafny-Rahbi B, Matejuk A, Grillon C, Kieda C. Why is the partial oxygen pressure of human tissues a crucial parameter? Small molecules and hypoxia. J Cell Mol Med. 2011; 15:1239–53. 10.1111/j.1582-4934.2011.01258.x 21251211 PMC4373326

[23] McKeown SR. Defining normoxia, physoxia and hypoxia in tumours-implications for treatment response. Br J Radiol. 2014; 87:20130676. 10.1259/bjr.20130676 24588669 PMC4064601

[24] McCarty G, Loeb DM. Hypoxia-sensitive epigenetic regulation of an antisense-oriented lncRNA controls WT1 expression in myeloid leukemia cells. PLoS One. 2015; 10:e0119837. 10.1371/journal.pone.0119837 25794157 PMC4368825

[25] Zhu T, Zheng SC, Paul DS, Horvath S, Teschendorff AE. Cell and tissue type independent age-associated DNA methylation changes are not rare but common. Aging (Albany NY). 2018; 10:3541–57. 10.18632/aging.101666 30482885 PMC6286821

[26] Naue J, Hoefsloot HC, Mook OR, Rijlaarsdam-Hoekstra L, van der Zwalm MC, Henneman P, Kloosterman AD, Verschure PJ. Chronological age prediction based on DNA methylation: massive parallel sequencing and random forest regression. Forensic Sci Int Genet. 2017; 31:19–28. 10.1016/j.fsigen.2017.07.015 28841467

[27] Maegawa S, Lu Y, Tahara T, Lee JT, Madzo J, Liang S, Jelinek J, Colman RJ, Issa JJ. Caloric restriction delays age-related methylation drift. Nat Commun. 2017; 8:539. 10.1038/s41467-017-00607-3 28912502 PMC5599616

[28] Wezyk M, Spólnicka M, Pośpiech E, Pepłońska B, Zbieć-Piekarska R, Ilkowski J, Styczyńska M, Barczak A, Zboch M, Filipek-Gliszczynska A, Skrzypczak M, Ginalski K, Kabza M, et al. Hypermethylation of TRIM59 and KLF14 Influences Cell Death Signaling in Familial Alzheimer’s Disease. Oxid Med Cell Longev. 2018; 2018:6918797. 10.1155/2018/6918797 29849909 PMC5904768

[29] Ciccarone F, Malavolta M, Calabrese R, Guastafierro T, Bacalini MG, Reale A, Franceschi C, Capri M, Hervonen A, Hurme M, Grubeck-Loebenstein B, Koller B, Bernhardt J, et al. Age-dependent expression of DNMT1 and DNMT3B in PBMCs from a large European population enrolled in the MARK-AGE study. Aging Cell. 2016; 15:755–65. 10.1111/acel.12485 27169697 PMC4933658

[30] Horvath S. Erratum to: DNA methylation age of human tissues and cell types. Genome Biol. 2015; 16:96. 10.1186/s13059-015-0649-6 25968125 PMC4427927

[31] DeCaprio JA, Ludlow JW, Figge J, Shew JY, Huang CM, Lee WH, Marsilio E, Paucha E, Livingston DM. SV40 large tumor antigen forms a specific complex with the product of the retinoblastoma susceptibility gene. Cell. 1988; 54:275–83. 10.1016/0092-8674(88)90559-4 2839300

[32] Ahuja D, Sáenz-Robles MT, Pipas JM. SV40 large T antigen targets multiple cellular pathways to elicit cellular transformation. Oncogene. 2005; 24:7729–45. 10.1038/sj.onc.1209046 16299533

[33] Triche TJ Jr, Weisenberger DJ, Van Den Berg D, Laird PW, Siegmund KD. Low-level processing of Illumina Infinium DNA Methylation BeadArrays. Nucleic Acids Res. 2013; 41:e90. 10.1093/nar/gkt090 23476028 PMC3627582

[34] Aryee MJ, Jaffe AE, Corrada-Bravo H, Ladd-Acosta C, Feinberg AP, Hansen KD, Irizarry RA. Minfi: a flexible and comprehensive Bioconductor package for the analysis of Infinium DNA methylation microarrays. Bioinformatics. 2014; 30:1363–69. 10.1093/bioinformatics/btu049 24478339 PMC4016708

