# Supplementary figures and images for: Epigenetic clock analysis of human fibroblasts in vitro: effects of hypoxia, donor age, and expression of hTERT and SV40 largeT
Source: Aging (Albany NY). 2019 May 21;11(10):3012–22. doi: 10.18632/aging.101955 (PMC6555444; doi:10.18632/aging.101955)

SUPPLEMENTARY FIGURE

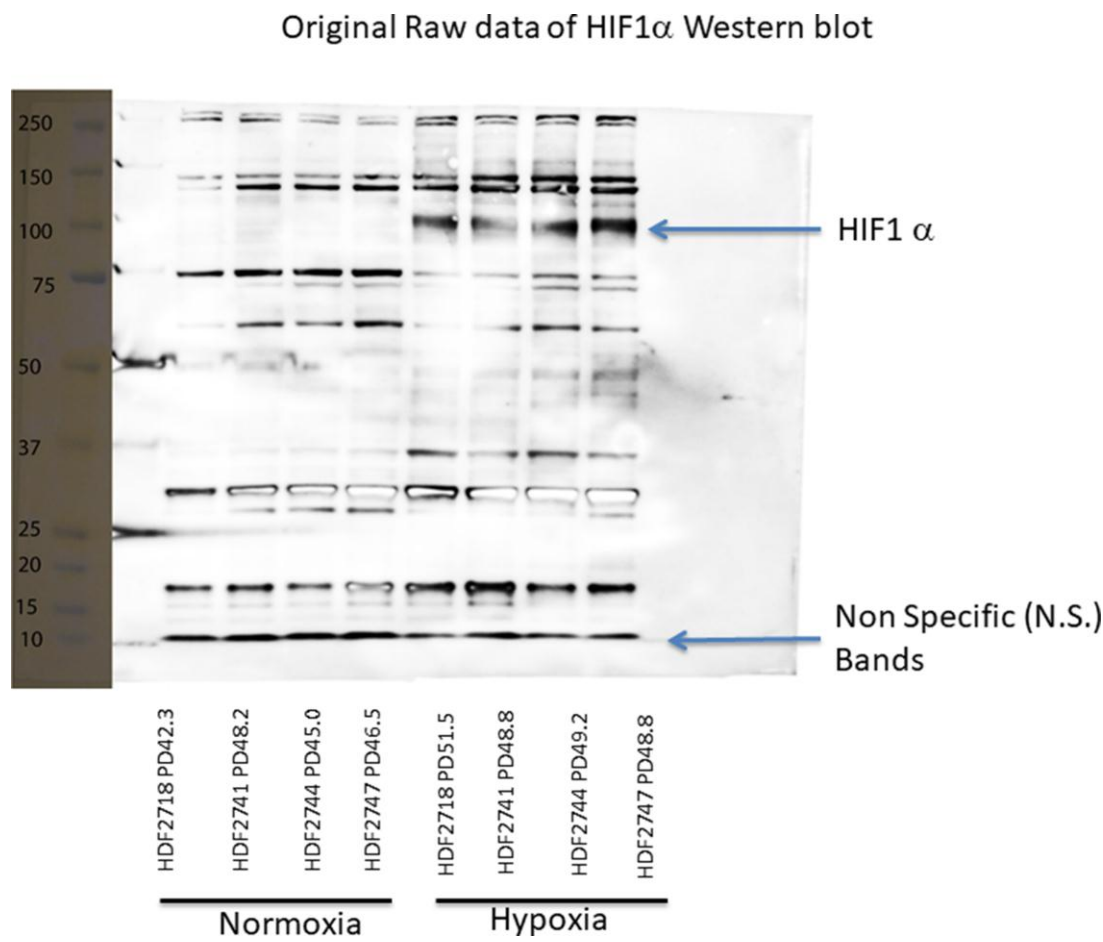

Supplementary Figure 1.

Supplement: Supplementary Figure [file aging-11-101955-s001.pdf]
